# Editorial: Food As Medicine

**DOI:** 10.3389/fnut.2024.1490232

**Published:** 2024-10-09

**Authors:** Andrea K. Boggild

**Affiliations:** ^1^Tropical Disease Unit, Toronto General Hospital, Toronto, ON, Canada; ^2^Department of Medicine, University of Toronto, Toronto, ON, Canada; ^3^Institute of Medical Science, Temerty Faculty of Medicine, University of Toronto, Toronto, ON, Canada

**Keywords:** Food As Medicine, nutrition, chronic diseases, dietary intervention, lifestyle intervention, micronutrients

“Let food be thy medicine”—a mantra famously attributed to Hippocrates—captures the central role of nutrition and dietary patterns in human health. Not only is the food we consume linked to prevention of diseases of dietary deficiency such as scurvy, pellagra, and Kwashiorkor, so too is it related to diseases of caloric abundance, such as type 2 diabetes, obesity, and hypertension. Moreover, patterns of food consumption are increasingly linked to restoration of health and maintenance of disease-free states following diagnoses such as cardiovascular disease, stroke, and cancer. Our ever-expanding knowledge of the human microbiome's role in health and disease continues to implicate patterns of food consumption to microbial diversity and function, and their impact on mood, cognitive status, and metabolic health. Never has the scientific examination of Hippocrates' famous tenet been more timely and needed. Food As Medicine is complementary to the field of lifestyle medicine, which promotes health behavior change across six domains, including nutrition, exercise, sleep, stress, or substance use/exposure to prevent, treat, and potentially reverse lifestyle-related, chronic disease.

In this *Food As Medicine* Research Topic, we holistically examine the role of nutrition and dietary patterns on health and disease states at the individual, community, and population levels. We aimed to synthesize the current state of knowledge in the Food As Medicine arena, and highlight effective nutrition-based interventions while also elucidating gaps in our understanding and identifying scientific strategies to close them. The methods highlighted in this Research Topic are representative across evidentiary pathways and include illustrative cases, systematic reviews, cohort studies, intervention trials and protocols, and expert reviews and commentary.

One core goal for the *Food As Medicine* Research Topic was to showcase high-quality, original research demonstrating improvements in intermediate health metrics or hard endpoints based on nutrition interventions administered to individuals with current illness. The Research Topic has additionally proposed approaches to operationalize and validate effective tools and strategies at a population and health care systems level.

Of the 23 papers ultimately accepted and published, two report on Food As Medicine correlates in individual patients (Goldner and Staffier) or small cohorts (Rowley et al.); six are clinical trials reporting on Food As Medicine interventions for specific chronic diseases such as: overweight and obesity (Kelly et al.; Kirbach et al.; Baden et al.), hypertension (Sijangga et al.), metastatic breast cancer (Lee et al.), and type 2 diabetes (Koh et al.); six are feasibility studies or other programmatic evaluations of lifestyle-based nutrition interventions (Albert et al.; Friedman et al.; Nyong et al.; Folta et al.; Staffier et al.; Karlsen et al.); three are topical reviews (Rosenfeld et al.; Qu et al.; Merlo et al.); one is a methods paper (Klowak and Boggild); three are knowledge and nutrition assessment studies among populations targeted for dietary interventions (Bai et al.; Li et al.; Quach et al.); and two are perspectives (Ayoob; McDougall).

Forty-eight percent of articles featured in the *Food As Medicine* Research Topic reported findings in equity-deserving groups including: Individuals who are deaf and hard of hearing (Friedman et al.); pregnant women (Rowley et al.); members of a US Latina/o/x community (Koh et al.); members of a US African American Faith-based community (Nyong et al.); members of a US Filipino community (Sijangga et al.); children (Li et al.); individuals of low socioeconomic status (Folta et al.; Bai et al.; Klowak and Boggild); and women with highly gendered chronic diseases such as systemic lupus erythematosus (Goldner and Staffier) and metastatic breast cancer (Lee et al.).

The specific organ systems addressed by research featured in the *Food As Medicine* Research Topic include: brain and neuronal health (Merlo et al.; Klowak and Boggild); cardiovascular and cardiometabolic health (Albert et al.; Rowley et al.; Koh et al.); periodontal health (Qu et al.); and gastrointestinal health (Quach et al.).

In Goldner and Staffier, three patients with systemic lupus erythematosus and Sjogren's syndrome followed a dietary protocol comprised predominantly of raw foods and emphasizing leafy greens, cruciferous vegetables, omega-3 polyunsaturated fatty acids, and water, and over the course of 4 weeks noted dramatic improvement in clinical symptoms, with complete symptom resolution out to many years of follow-up. Such anecdotal evidence for dietary control of autoimmune diseases provides a tantalizing foundation on which to base further prospective studies aiming to disentangle the influence of lifestyle on inflammatory processes.

In Rowley et al., 51 pregnant women were stratified according to their personal alignment with a Mediterranean diet, which was correlated to urinary and serum metabolites and inflammatory biomarkers at 36-weeks gestation. Demonstrable reductions in inflammatory biomarkers were notable in the women with high alignment to the Mediterranean diet compared to those with low alignment characterized by greater consumption of red meat and lower intake of fruits and vegetables. Extending such biological findings to health and developmental outcomes in both pregnant women and their infants warrants future investigation.

Among the six clinical trials reporting on Food As Medicine interventions for specific chronic diseases, Kelly et al. report on a 16-week plant-predominant fiber-rich nutrition program that was delivered to over 4,000 employees at 72 different employers across the Southwest United States over a 3-year period. This “Full Plate Living” program translated to an average 3.28 kg weight loss among the >60% of participants who lost weight during the trial, and drastically improved the daily intake of health-promoting fiber-rich foods including fruits (2.45 servings/day), vegetables (2.99 servings/day), beans (1.03 servings/day), and total fiber composites (9.07 servings/day) compared to those participants who did not lose weight. Such findings validate the role of dietary fiber in the lifestyle management of overweight and obesity.

Kirbach et al. report on the implementation and successive iteration via Plan-Do-Study-Act (PDSA) cycles of the Supervised Lifestyle Integrative Medicine (SLIM) program, which is a virtually delivered, lifestyle medicine focused shared medical appointment (SMA) program, situated within a weight management clinic of a larger health system, that served 172 participants over 2 years. The ultimate model combines one-on-one and group interactions over 12-weeks with a dedicated team including an Obesity Medicine and Lifestyle Medicine physician, registered dietician, health coach, and preventive medicine resident.

In their pilot study examining the feasibility of a 3-month lifestyle modification program based on a “Teaching Kitchen” in Japan, Baden et al. report that among 24 participants with obesity, significant post-intervention improvements were noted in weight, body mass index, diastolic blood pressure, body fat mass, and consumption of total fat and dietary sodium. Health related quality of life indices were further improved, notably on measures of bodily pain, general health, vitality, and mental wellness. Given high program completion rates and the aforementioned improvements in biometry, the program was deemed feasible.

In order to address the gap in culturally-relevant lifestyle options for blood pressure management currently available to the Filipino community, Sijangga et al. report on the development of a cookbook using participatory methods and design thinking, utilizing input from five Filipino culinary experts and a Registered Dietitian. Among 20 Filipinx participants with self-reported, physician-diagnosed hypertension included in the pilot test of the cookbook, evidence of its acceptability and feasibility emerged, with participants reporting that the recipes, nutrition labels, illustrations, and cultural aspects of the cookbook increased their motivation to pursue dietary changes aimed at reducing blood pressure, notably reducing dietary sodium intake.

Lee et al. report the findings of an 8-week whole foods plant-based (WFPB) dietary RCT in metastatic breast cancer patients on stable therapy whose intake of isoflavones and both omega-3 and omega-6 polyunsaturated fatty acids (PUFAs) were assessed both pre- and post-intervention. In the WFPB group, total daily intake of isoflavones increased from a mean of 0.8–14.5 mg/day (*p* < 0.0001), and the *n*-6:*n*-3 ratio of PUFAs decreased from a mean of 9.3–3.7 (*p* < 0.0001), providing evidence that even short courses of the WFPB diet intervention translates into meaningful changes in serum biomarkers of healthful nutrition. As with the Rowley et al. cohort, extension of the biological findings to both intermediate and long-term health outcomes through future prospective studies is warranted.

Koh et al. report on the lessons learned during the design, implementation, and evaluation of a remotely-accessible, community-based, nurse-led, culturally-tailored WFPB culinary intervention to reduce type 2 diabetes risk among Latina/o/x adults. Through their mixed-methods quasi-experimental study involving both pre- and post-evaluation and comprised of questionnaires, culinary instruction, biometry, and focus groups, the authors identify for prioritization: improved accessibility and engagement in minoritized and/or underserved communities; quality assurance and service delivery along the supply chain; sustainable study design; and interventions that are remotely accessible.

In the six studies of program feasibility or other programmatic evaluations of lifestyle-based nutrition interventions, findings were equally compelling. Albert et al. report on intermediate health outcomes—including hemoglobin A1c (HbA1c), blood pressure, body weight, and serum cholesterol—of 173 participants in a Plant-Based Lifestyle Medicine Program piloted in a New York City safety-net hospital. Over the 1-year program, the participant cohort achieved statistically significant improvements in body weight, HbA1c, and diastolic blood pressure. Among those with prediabetes, overweight or obesity, significant improvements in weight were achieved, while those with type 2 diabetes experienced significant improvements in both weight and HbA1c. Similarly, participants with hypertension achieved significant reductions in diastolic blood pressure and weight. Extending these findings to long-term health outcomes such as microvascular complications of diabetes, myocardial infarction, and cancer in larger prospective cohorts will provide even further compelling evidence of Food As Medicine for obesity and type 2 diabetes.

Understanding that persons who are deaf and hard of hearing (DHH) are at risk of developing chronic preventable diseases and have worse health outcomes when they do, Friedman et al. report on the Rochester Lifestyle Medicine Institute's adapted, online, Zoom-based, medically-facilitated “15-Day Whole-Food Plant-Based (WFPB) Jumpstart” program designed to provide this at-risk cohort the knowledge, skills, and support to undertake health-related dietary improvements. All participants lost weight, had decreases in pulse and systolic blood pressure, and reduced their total and LDL cholesterol. Participants further reported increased energy, quality sleep, and mood, and noted commensurate improvements in their knowledge and skills owing to the program.

Understanding that the African American population is disproportionately affected by many of the leading causes of preventable death, including hypertension, obesity, heart disease, stroke, and type 2 diabetes, Nyong et al. also report on the Rochester Lifestyle Medicine Institute's “15-Day Whole-Food Plant-Based (WFPB) Jumpstart” program delivered to participants recruited via a network of predominantly African American churches throughout the State of Illinois. Pre- and post-program metabolic screening of weight, vital signs, blood sugar, and cholesterol were undertaken and demonstrated: an average weight loss among 21 participants of 5.8 pounds; an average 10-point systolic blood pressure reduction; and an average 37-point total cholesterol decrease. Participants reportedly ate more vegetables, greens, fruit, whole grains, and legumes during the program and also reduced their consumption of meat, eggs and dairy, added fat, processed foods, and high-fat plant foods, thus correlating positive dietary changes to dramatic improvements in health-related biometrics.

In their qualitative case comparison study, Folta et al. report on the adoption and implementation factors related to a produce prescription program designed to close disparities in diet quality and diet-related chronic disease for persons of lower socioeconomic status. Factors such as incorporation into clinic workflow and fit with operations were raised by implementing staff as key facilitators to adoption, while the need for extra time and sustainability were cited as threats to long-term implementation.

In their online, cross-sectional survey of >6,000 individuals who downloaded the American College of Lifestyle Medicine's complimentary “Culinary Medicine Curriculum” (CMC), Staffier et al. report that while 70% of enrolled participants neither led nor created any specific sessions related to the CMC, the 30% who did, did so across clinical settings including academia, clinical establishments, and coaching practices, and represented a range of disciplines within the lifestyle medicine arena including physicians, registered dietitian nutritionists, and chefs. Future studies investigating the impact of a CMC on intermediate and long-term health outcomes for patients and clients will be a valuable extension of this work.

In their description of the development and pilot testing of a brief, dietary screener to assess the proportion of whole, unrefined plant-based foods and water relative to total food and beverage consumption, Karlsen et al. report that among 539 lifestyle medicine practitioners surveyed, >60% assess diet quality informally, and 80% report facing barriers to dietary screening in the clinical setting. As such, the newly developed screener, which consists of a 27-item diet assessment tool, can serve as a successful addition to the lifestyle medicine practitioners' clinical armamentarium.

In the first of three knowledge and nutrition assessment studies among populations targeted for dietary interventions, Quach et al. conducted a cross-sectional survey among >4,000 Vietnamese adults, over half of whom reported ongoing gastroesophageal reflux symptoms (GERS) that were troublesome. Factors associated with troublesome GERS included: eating beyond fullness, stress, insomnia, and consumption of particular trigger foods such as greasy foods, sour and/or spicy soups, citrus fruits, and carbonated soft drinks. This novel study of dietary and lifestyle factors associated with troublesome GERS in Vietnamese adults has laid the groundwork for future studies of prevention and effective lifestyle interventions.

In their exploratory analysis of nutritional knowledge, health literacy and dietary behaviors in 400 rural Chinese residents, Bai et al. report that the mean total nutritional knowledge score was 7.19 out of a maximum score of 13, indicating that declarative nutrition knowledge in this population is suboptimal. Moreover, dietary behaviors, particularly consumption of fruits, beans, and vegetables were equally poor, with male, elderly, low-income, unmarried, and persons with low-education at greatest risk of inadequate nutrition knowledge and behaviors. Such data are important to inform the design and implementation of strategies to address these disparities in health knowledge, attitude, and behaviors.

Li et al. conducted individual and small group interviews with 23 adult female caregivers of young, pre-school aged children who participated in a Texas-based produce prescription program during the COVID-19 pandemic, and noted that >80% of caregivers were Hispanic/Latino and >40% of participating families had three or more children. Thematic analysis enabled feedback to emerge around program logistics including ease of use, participant satisfaction, and desire for additional store bought options; as well as program impact including the improved ability to purchase produce, the utility of nutrition education provided, and continued challenges with preparation of produce for “picky eaters” and very young children. Understanding the facilitators and barriers to adequate produce consumption in young children, particularly those who have intersecting vulnerabilities such as neurodivergence, is critical to developing policy and programs in pediatric health.

In their description of a prospective, randomized controlled single-blind, multicenter interventional trial for a WFPB dietary intervention for the chronic neuropathic pain of leprosy, Klowak and Boggild identify that diets rich in plant-based macro- and micronutrients are likely to improve physiological and metabolic neuronal health, reduce systemic inflammation, and enhance immune responsiveness to environmental neurotoxic factors. Given that type 2 diabetes is an exceptionally common comorbidity of leprosy, the authors hypothesize that WFPB diets will mitigate progression and severity of peripheral neuropathic pain and potentially reduce the adverse events related to standard corticosteroid treatment of leprosy reactions due to their inherently anti-inflammatory nature.

Among the three topical reviews, in their scoping review of 95 articles (54% longitudinal, 37% cross-sectional, and 9% case–control) with a median sample size of >3,500 participants investigating how the quality of plant-based dietary patterns might be associated with health outcomes, Rosenfeld et al. report that higher healthful plant-based dietary index levels were associated with favorable health outcomes in over a third of comparisons, notably for obesity, mortality, diabetes, cardiovascular disease, and psychiatric disorders. On the other hand, higher levels of unhealthful plant-based dietary indices were associated with unfavorable health outcomes in a third of comparisons. The scoping review underscores how a focus on the quality of healthful diets is important to incorporate into nutrition guidance.

In their exploration of “Medicine food homology” (MFH), which acknowledges that traditional natural products have both nutritional and medicinal benefits, Qu et al. summarize the existing state of knowledge around the MFH plants that can prevent and treat periodontitis. Mechanistically, numerous MFH plant metabolites and extracts have demonstrable antibacterial action against periodontal infections. Moreover, MFH plants have been found to inhibit host inflammatory responses and bone resorption in periodontal infections. Given the severe quality of life implications of periodontal infections, MFH as a discipline has important applications in the lifestyle medicine arena.

In their review of the brain-gut-microbiota (BGM) system, Merlo et al. highlight its significant influence on cognitive processing, mood regulation and dysregulation, neuroplasticity, and other metrics of mental and neuronal health. Given that poor nutrition is linked to increased risks to brain health, mental health, and psychological functioning, the BGM system represents an important target for both preventive and therapeutic lifestyle-based interventions.

In the two perspective pieces published, Ayoob provides key insights into the concept of carbohydrate interchangeability and whether or not starchy vegetables can be considered interchangeable with grains. Given the loss of key micronutrients with interchangeability as ascertained through menu modeling analyses, the author provides an argument to categorize starchy vegetables and grains separately. Finally, McDougall argues that the dietary patterns that best support human health and that of planet Earth are underpinned by traditional starchy staples including but not limited to: rice in Asia, corn in Central and South America, potatoes in the Andes, and wheat and barley throughout the Middle East. The author further supports his argument that optimal human health requires a shift away from destructive animal-food based-diets to those centered around plant-based foods.

In conclusion, the *Food As Medicine* Research Topic provides new insights and novel data accrued through primary research into how dietary patterns, practices, and adjacent technologies can serve to improve metabolic markers of disease and biometric outcomes across different communities, life stages, and states of health and disease. Extending the data presented herein to studies of long-term health outcomes will fill existing knowledge gaps of how dietary and nutrition interventions may best be used in clinical and therapeutic environments, and as preventive public health programming.

